# Comparison of pre-procedural anxiety and depression scores for patients undergoing chorion villus sampling and amniocentesis: An alternative perspective on prenatal invasive techniques

**DOI:** 10.12669/pjms.315.7477

**Published:** 2015

**Authors:** Cem Yasar Sanhal, Inanc Mendilcioglu, Murat Ozekinci, Mehmet Simsek, Selen Bozkurt

**Affiliations:** 1 Cem Yasar Sanhal, MD. Dr. Zekai Tahir Burak Women’s Health Education and Research Hospital, Ankara, Turkey; 2 Inanc Mendilcioglu, Professor, Gynecology and Obstetrics Department, Akdeniz University Faculty of Medicine, Antalya, Turkey; 3 Murat Ozekinci, Assistant Professor, Gynecology and Obstetrics Department, Akdeniz University Faculty of Medicine, Antalya, Turkey; 4 Mehmet Simsek, Professor, Gynecology and Obstetrics Department, Akdeniz University Faculty of Medicine, Antalya, Turkey; 5 Selen Bozkurt, Department of Biostatistics and Medical Informatics, Akdeniz University Faculty of Medicine, Antalya, Turkey

**Keywords:** Amniocentesis, Chorion villus sampling, Anxiety, Depression, Beck depression, Spielberger State-Trait Anxiety Inventory

## Abstract

**Objective:**

To compare the pre-procedural anxiety and depression levels of patients undergoing chorion villus sampling (CVS) and amniocentesis (AC).

**Methods:**

Patients referred to our department for fetal karyotype analysis with a positive first or second trimester screening test for aneuploidy between January 2013 to June 2015 were included. CVS and AC procedures were performed in patients with gestation periods of between 11-14 and 16-20 weeks, respectively. Anxiety was evaluated using the Spielberger State-Trait Anxiety Inventory (STAI), and depression was assessed using the Beck Depression Inventory II (BDI-II).

**Results:**

A total of 1,400 patients were included. Compared to first trimester controls, patients undergoing CVS had significantly higher STAI-state and BDI-II results. Likewise, patients undergoing AC had higher STAI-state and BDI-II scores than controls in the second trimester. In terms of STAI-trait results, no difference was found between the groups. Our results also showed that, compared to AC group, patients undergoing CVS had similar STAI-state, STAI-trait and but higher BDI-II scores.

**Conclusion:**

We conclude that evaluating the stress and depression levels of these patients should be one of the routine procedures in pregnancy follow-up.

## INTRODUCTION

Pregnancy itself is one of the most stressful and depressing periods in a woman’slife.^1^ The possibility of miscarriage or other perinatal problems is the main cause of this condition. Prenatal diagnosis of fetal abnormalities, particularly fetal aneuploidies, are of major interest to contemporary perinatal medicine. It is clear that all approaches, including invasive methods, are usually used for confirming the precise diagnosis of an abnormality.

The procedures used for fetal genetic analysis are divided according to the gestational time and the indicated tissues.^2^,^3^ The earliest procedure is chorion villus sampling (CVS), which is typically performed at a gestation period of between 10 and 14 weeks. The alternative method – amniocentesis (AC) – is usually performed after 15 weeks. The advantage of CVS is early diagnosis, while the benefits of AC are less placental mosaicism and a slightly lower rate of procedure related abortion.^4^,^5^

Although the relations between maternal stress, anxiety and perinatal handicaps such as early labor onset, low birth weight, and poor fetal and child development have been reported, still very little attention is being paid among obstetricians on the detection, significance and treatment of anxiety and depression, particularly in daily routine practice.^6^-^8^

This issue also relates to all professionals who perform invasive procedures such as CVS or AC. Generally, a successful and uncomplicated invasive procedure is the main goal for the obstetrician. Very few concern themselves with the psychological state of the patient. Nevertheless, patients suffering from severe anxiety and/or depression may need psychiatric support.

We performed this study in order to compare the pre-procedural anxiety and depression levels of patients undergoing CVS or AC.

## METHODS

The protocol of this study was approved by the ethics committee for human studies at our university (number: 04.09.2012/257), and informed consent was obtained from all participants before the study was conducted between January 2012 and June 2015.

All patients referred to our perinatology department for fetal karyotype analysis with a positive first or second trimester screening test for aneuploidy were recruited for this case-control study. CVS and AC procedures were performed in patients with gestation periods of between 11-14 and 16-20 weeks, respectively. A complete fetal sonographic examination was then performed to document other structural anomalies, and patients with a fetal anomaly and/or multiple pregnancies were excluded.

Anxiety was measured prior to the procedure using the STAI, and depressive symptoms were assessed using the BDI-II, again before the procedure. Twenty items were considered in assessing the trait measure, which reflects more stable characteristics – an anxious personality, for example. The other 20 items assess the state measure, which reflects transient characteristics such as anxiety disorders. The STAI questionnaire rates responses on a four-point intensity scale ranging from ‘not at all’ to ‘very much’. The range of scores is 20-80. Higher scores are positively correlated with higher levels of anxiety.^9^,^10^ The Turkish version of the STAI questionnaire used in this study has been validated in Turkish population.^11^

The BDI-II is a 21-item self-reporting questionnaire that assesses the severity of depression. Individuals are asked to rate themselves on a spectrum of 0-3 (0= least, 3= most) with a score range of 0-63. The cut-offs are 0-13, minimal depression; 14-19, mild depression; 20-28, moderate depression; and 29-63, severe depression. The total score is the sum of all the items. The Turkish version of the BDI-II used in this study has been validated in Turkish populations.^12^,^13^

All patients referred for first or second trimester screening tests for aneuploidy were informed about the study and invited to complete the above inventories to be included in the control group.

Continuous variables are presented as mean±standard deviation, while categorical variables are given as percentages. The Kolmogorov–Smirnov test was used to detect normal distribution. Given the non-parametric distribution of the groups, statistical analysis of the mean ranks between the groups was made using the Mann-Whitney U test. Categorical data was analysed by Pearson Chi-square test and Chi-square for trend. In addition Spearman correlation analysis was used to assess correlation among variables. Analyses were performed with PASW 18 (SPSS/IBM, Chicago, IL, USA) software and two-tailed P value less than 0.05 was considered statistically significant.

## RESULTS

A total of 1,400 patients were included in the study. The numbers of patients undergoing CVS and AC procedures were 220 and 480, respectively. The control group consisted of 220 patients in the first trimester (controls for the CVS group) and 480 patients in the second trimester (controls for the AC group).

The characteristics of the groups are shown in Table-I. There were no statistically significant differences between CVS vs. first trimester controls, AC vs. second trimester controls, and CVS vs. AC with respect to maternal age, partner’s age, duration of marriage, body mass index (BMI), monthly income, occupation, healthy child from a previous pregnancy, maternal education level, and place of residence.

**Table-I:** Patient characteristics of groups.

	CVS (n=220)	1st trcont (n=220)	AC (n=480)	2nd trcont (n=480)
Age (years)	29,9 ± 5,7	29,1 ± 5,1	30,7 ± 6,5	28,5 ± 5,3
Partner’s age (years)	32,6 ± 6,3	32,5 ± 5,1	33,6 ± 6,7	31,3 ± 5,7
Duration of marriage (years)	6,9 ± 4,9	6,3 ± 4,3	6,7 ± 5,8	6,0 ± 4,6
BMI	24,1 ± 4,0	24,7 ± 4,2	25,1 ± 4,2	24,6 ± 3,9
*Occupation (%)*				
Yes	30,0	27,7	27,9	29,8
No	70,0	72,3	72,1	70,2
Income (TL)	1540± 937	1403±778	1479±839	1450±803
*Healthy child from a previous pregnancy (%)*				
Yes	42,3	40,9	37,9	38,5
No child	57,7	59,1	62,1	61,5
*Educational status (%)*				
≤5 years	6,4	5,5	5,6	5,4
6-12 years	80,0	77,3	81,9	81,3
≥12 years	13,6	17,3	12,5	13,3
*Place of residence (%)*				
City	71,8	68,2	73,5	72,9
Village	28,2	31,8	26,5	29,1

The comparison of STAI and BDI-II scores between groups are sown in Table-II. Minimal, mild, moderate and severe depression is represented by BDI-II-0, BDI-II-1, BDI-II-2, BDI-II-3 and BDI-II-4, respectively. Compared to first trimester controls, patients undergoing CVS had significantly higher STAI-state and BDI-II results. Likewise, patients undergoing AC had higher STAI-state and BDI-II scores than controls in the second trimester. In terms of STAI-trait results, no difference was found between the groups. Our results also showed that, compared to AC group, patients undergoing CVS had similar STAI-state, STAI-trait and but higher BDI-II scores.

**Table-II:** Comparison of STAI and BDI-II scores between groups.

	CVS (n=220)	1st trcont (n=220)	AC (n=480)	2nd trcont (n=480)	P
STAI-State	48,60 ± 7,78	36,00 ± 8,44	47,95 ± 8,88	36,05 ± 7,07	CVS vs. 1st tr^a^
					AS vs. 2nd tr^a^
					CVS vs. AS
STAI-Trait	43,78 ± 5,40	42,79 ± 7,25	44,93 ± 7,47	43,82 ± 7,09	CVS vs. 1st tr
					AS vs. 2nd tr
					CVS vs. AS
BDI-II –	47,7 %	79,1 %	45,8 %	80 %	CVS vs. 1st tr^b^
BDI-II – 1	25,0 %	10,0 %	26,9 %	16,3 %	AS vs. 2nd tr^b^
BDI-II – 2	15,9 %	9,1 %	20,8 %	3,5 %	CVS vs. AS^b^
BDI-II – 3	11,4 %	1,8 %	6,5 %	0,2 %	

a denotes significant difference (Mann-Whitney test)

b denotes significant difference (Chi-square for trend test)

Minimal, mild, moderate & severe depression is represented by BDI-II-0, BDI-II-1, BDI-II-2, BDI-II-3 & BDI-II-4, respectively.

Finally, no significant effects of maternal age, occupational status, a healthy child from a previous pregnancy, level of education and place of residence were determined on STAI-state, STAI-trait and BDI-II scores.

## DISCUSSION

In this prospective study, we surveyed pregnant women undergoing a prenatal invasive procedure (CVS or AC) after an abnormal first or second trimester screening test for aneuploidy, and compared them to controls. We found that both procedures were associated with higher scores for state anxiety and depression. Moreover, our results revealed that patients undergoing CVS had higher scores for depression than patients undergoing AC.

Comparison of invasive prenatal diagnosis techniques (CVS and AC) and invasive fetal therapy was performed as part of a previous study designed to examine the psychosocial impacts of these methods.^14^ Results showed that pregnant women awaiting invasive prenatal diagnosis and fetal therapy experienced higher levels of state anxiety than women undergoing non-invasive procedures. In the same study, it was also reported that the depression scores of women in CVS and AC groups did not differ from controls, that was in line with some previous studies.^15^,^16^ Some other studies declared that the level of anxiety was significantly higher in women undergoing CVS than AC.^17^,^18^ Anxiety levels were even higher when the indication for the procedure was fetal structural abnormality.^17^ Although the authors used STAI and BDI to examine anxiety and depression in the study comparing fetal invasive therapy and invasive prenatal diagnosis techniques, we feel that the relatively small number of participants, discrete scales, and distinct patient characteristics were some of the reasons for conflicting results with our study. The present study involved a larger cluster of participants. To eliminate the potential effects of other factors, we only distinguished patients with an abnormal first or second trimester screening test, excluding pregnant women with a fetal abnormality and any previous fetus or child with any chromosomal, genetic or structural abnormality. However, further studies should be conducted in order to enlighten the main cause of the difference between CVS and AC with respect to depression scores, which were detected in our study.

Women are at an increased risk of developing anxiety and depressive disorders during the perinatal period.^19^,^20^ The possibility of being faced with a fetal problem is a major cause of this situation. However, in a previous trial, it was asserted that the mean levels of depression and anxiety were similar in women awaiting a prenatal ultrasound evaluation or procedures such as CVS or AC.^15^ Moreover, the individual’s experience of prenatal diagnosis was shown to be stressful in and of itself, with stress levels not determined by the invasiveness of the procedure.^15^ Our control groups included patients in the first trimester (controls for CVS) and second trimester (controls for AC) who were offered a screening test (combined test for first trimester, quadruple test for second trimester) for aneuploidy. In this respect, our study differed from those cited above. We believe that CVS or AC ascribes further complexity to the experiences of pregnant women in regard to state anxiety and stress. In addition, our findings did not reveal any differences in anxiety trait scores. This result might be the outcome of the characteristics of the STAI-trait test which measures the general feeling and properties of the tested population.

Antenatal depression, as well as both state and personality trait anxiety, have been shown to be some of the strongest risk factors associated with postpartum depression.^21^,^22^ Approximately half of the women fulfilling the criteria for postpartum depression exhibited symptoms of the disorder during pregnancy.^23^ However, there is still lack of universal concern regarding the emotional state of pregnant women during the diagnostic continuum. Our findings – 11.4% for severe depression in the CVS group, and 6.5% in the AC group – indicate that both of these are far higher than the average rate of physician-diagnosed gestational major depression (0.8%).^24^ The reported data indicating the higher risks of women with depression and anxiety regarding miscarriage, perinatal death and decisions to terminate a pregnancy if prescribed psychotropic medication during early pregnancy further highlighted the importance of depression and anxiety.^25^ Hence, patients undergoing an invasive prenatal procedure should also be examined to determine if they require psychiatric support.

Perinatal diagnostic technologies are currently at their highest ever level. We feel that a similar level of attention should be given to the evaluation, and if necessary treatment, of stress and depression in patients undergoing invasive prenatal procedures. Such measures would serve to lessen the potential associated negative influences on perinatal outcomes.

### Limitations of the study

As this study aimed at evaluating the pre-procedural anxiety and depression levels of the patients about to undergo CVS or AC, this was the largest of its type. The case-control design depending on the type of self-reporting questionnaire is hence a possible limitation of our study. Patient referral to a psychiatry clinic would also provide more valuable data on anxiety and depression levels, particularly for those patients with higher scores. Finally, revealing the long-term perinatal effects of high anxiety and depression levels would probably disclose more conclusive evidence.
